# The JAK/STAT Pathway Is Involved in Synaptic Plasticity

**DOI:** 10.1016/j.neuron.2011.11.024

**Published:** 2012-01-26

**Authors:** Céline S. Nicolas, Stéphane Peineau, Mascia Amici, Zsolt Csaba, Assia Fafouri, Charlotte Javalet, Valerie J. Collett, Lars Hildebrandt, Gillian Seaton, Sun-Lim Choi, Su-Eon Sim, Clarrisa Bradley, Kyungmin Lee, Min Zhuo, Bong-Kiun Kaang, Pierre Gressens, Pascal Dournaud, Stephen M. Fitzjohn, Zuner A. Bortolotto, Kwangwook Cho, Graham L. Collingridge

**Affiliations:** 1 MRC Centre for Synaptic Plasticity, School of Physiology and Pharmacology, Medical Sciences Building, University Walk, Bristol BS8 1TD, UK; 2 INSERM, U676, Robert Debré Hospital, 48 bvd Sérurier, 75019 Paris, France; 3 Université Paris 7, Faculté de Médecine Denis-Diderot, 75019 Paris, France; 4 MRC Centre for Synaptic Plasticity, Henry Wellcome L.I.N.E., School of Clinical Sciences, Bristol BS1 3NY, UK; 5 Department of Brain and Cognitive Sciences, Seoul National University, Seoul 151747, Korea; 6 National Creative Research Initiative Center for Memory, Department of Biological Sciences, College of Natural Sciences, Seoul National University, Seoul 151747, Korea; 7 Department of Anatomy, School of Medicine, Kyungpook National University, 2-101 Dongin-Dong, Daegu, Korea

## Abstract

The Janus kinase (JAK)/signal transducer and activator of transcription (STAT) pathway is involved in many cellular processes, including cell growth and differentiation, immune functions and cancer. It is activated by various cytokines, growth factors, and protein tyrosine kinases (PTKs) and regulates the transcription of many genes. Of the four JAK isoforms and seven STAT isoforms known, JAK2 and STAT3 are highly expressed in the brain where they are present in the postsynaptic density (PSD). Here, we demonstrate a new neuronal function for the JAK/STAT pathway. Using a variety of complementary approaches, we show that the JAK/STAT pathway plays an essential role in the induction of NMDA-receptor dependent long-term depression (NMDAR-LTD) in the hippocampus. Therefore, in addition to established roles in cytokine signaling, the JAK/STAT pathway is involved in synaptic plasticity in the brain.

## Highlights

► Identification of a specific role of the JAK/STAT pathway in synaptic plasticity ► Activation of JAK2 and STAT3 is required for NMDAR-LTD ► Activation of STAT3 in dendrites and nucleus during NMDAR-LTD ► Nuclear translocation of STAT3 is not required for NMDAR-LTD

## Teaser

The authors demonstrate a new neuronal function for the JAK/STAT pathway in the induction of NMDA-receptor-dependent long-term depression (NMDAR-LTD) in the hippocampus.

## Introduction

The Janus kinases (JAKs) are a family of non-receptor protein tyrosine kinases (PTKs) that consists of four mammalian isoforms: JAK1, JAK2, JAK3, and TYK2. They are activated in a variety of different ways. In the canonical pathway, two JAK molecules bind to two receptors that have dimerized in response to ligand binding and the juxtaposed JAKs trans and/or autophosphorylate resulting in their activation (Yamaoka et al., 2004). This mode of activation applies, for example, to cytokine receptors, growth-hormone like receptors and the leptin receptor. Alternatively, JAKs may be activated following stimulation of G protein-coupled receptors (GPCRs), PTKs such as PYK2 (Frank et al., 2002) and/or via intracellular calcium changes (Frank et al., 2002; Lee et al., 2010). Once activated, JAKs phosphorylate and activate downstream targets. The best established downstream effector of JAK is the signal transducer and activator of transcription (STAT) family. Seven STAT isoforms, named STAT1 to STAT4, STAT5A, STAT5B, and STAT6, have been identified. Once phosphorylated by JAK, STATs dimerize and are translocated to the nucleus where they regulate the expression of many genes (Aaronson and Horvath, 2002; Levy and Darnell, 2002; Li, 2008).

The JAK/STAT pathway is involved in many physiological processes including those governing cell survival, proliferation, differentiation, development, and inflammation. There is increasing evidence that this pathway also has neuronal specific functions in the central nervous system (CNS). For example, the JAK/STAT pathway is involved in leptin-induced neuroprotection and in the control of food intake (Bjørbaek and Kahn, 2004; Tups, 2009). This pathway has also been linked to Alzheimer's disease and memory (Chiba et al., 2009a; Chiba et al., 2009b). In particular, it has been shown recently that mice injected with the JAK inhibitor AG490 have spatial working memory impairment (Chiba et al., 2009b).

The cellular and molecular mechanism by which the JAK/STAT pathway is involved in neuronal function is unknown. It has, however, been shown that JAK can regulate the expression or function of several neurotransmitter receptors, including γ-amino-butyric acid (GABA) (Lund et al., 2008), muscarinic acetylcholine (Chiba et al., 2009b), N-methyl-D-aspartate (NMDA) and α-amino-3-hydroxy-5-methyl-4-isoxazole propionic acid (AMPA) receptors (Mahmoud and Grover, 2006; Orellana et al., 2005; Xu et al., 2008). To investigate how the JAK/STAT pathway may function within the CNS we have investigated whether it has a role in synaptic plasticity, the family of processes that are widely believed to underlie memory formation in the CNS.

The two major forms of long-lasting synaptic plasticity in the mammalian brain—long-term potentiation (LTP) and long-term depression (LTD)—are characterized by a long-lasting increase or decrease in synaptic strength, respectively. The dominant forms of both LTP and LTD are triggered by the synaptic activation of one class of glutamate receptor, the NMDA receptor, and are expressed as alterations in synaptic transmission mediated by another class of glutamate receptor, the AMPA receptor (Collingridge et al., 2004; Collingridge et al., 2010). However, how the transient activation of NMDARs leads to these processes is not fully understood.

We find that pharmacological inhibition of JAK blocks the induction of NMDAR-LTD at CA1 synapses in the hippocampus. This effect is highly selective since the same treatment that completely prevents the induction of NMDAR-LTD has no effect on LTP, depotentiation or LTD induced by the activation of metabotropic glutamate receptors (mGluRs). We find that the JAK2 isoform is enriched at synapses and knockdown of JAK2 also blocks the induction of NMDAR-LTD. Furthermore, activation of NMDARs, either pharmacologically or by low-frequency stimulation (LFS), causes a transient activation of JAK2. We also present evidence that the effects of JAK2 in NMDAR-LTD are mediated via STAT3. Thus, pharmacological inhibition or knockdown of STAT3 also blocks the induction of NMDAR-LTD. Furthermore, LFS causes activation and nuclear translocation of STAT3. However, we find that the nuclear translocation of STAT3 is not required for NMDAR-LTD, suggesting that the effect of STAT3 during NMDAR-LTD occurs in the cytoplasm. In summary, we show that the JAK/STAT pathway has a key role in synaptic plasticity.

## Results

### The JAK Inhibitor AG490 Specifically Blocks the Induction of NMDAR-LTD

**Figure 1:**
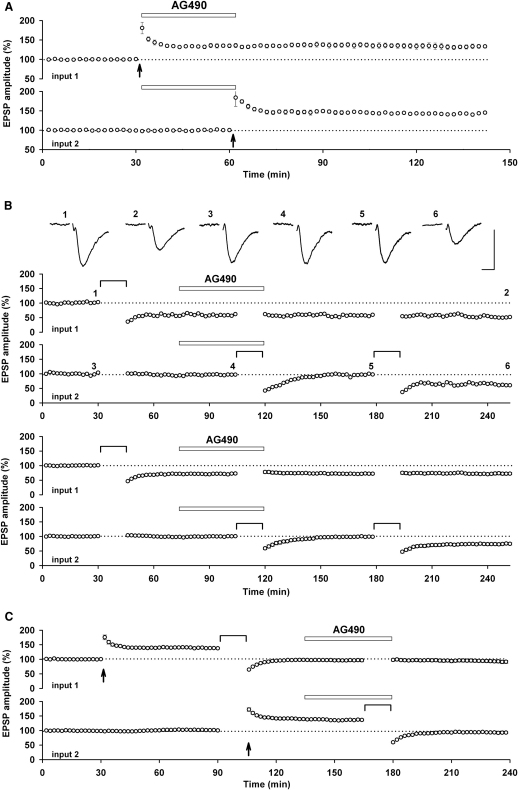
AG490 Blocks the Induction of NMDAR-LTD but Not LTP or Depotentiation (A) Pooled data (n = 4) showing that AG490 (10 μM) has no effect on baseline transmission or LTP. The duration of the drug application is indicated by the open rectangle and the timing of the LTP induction protocol is marked by an arrow. (B) A single example (top) and pooled data (bottom) showing that AG490 has no effect on pre-established LTD but blocks the induction of NMDAR-LTD in a reversible manner (n = 6). The NMDAR-LTD induction protocol, in this and subsequent figures, is marked by a horizontal line. The traces were obtained at the times indicated on the graph and are averages of 4 successive recordings. Calibration bars: 0.5 mV / 10 ms. (C) Pooled data (n = 6) showing that AG490 has no effect on depotentiation. In all experiments, two inputs were stimulated alternately and responses are averages of four successive recordings.

We first investigated the role of JAK in synaptic transmission and synaptic plasticity in the Schaffer collateral-commissural pathway (Figure 1). For the initial set of experiments we used extracellular recording in acutely prepared rat hippocampal slices and stimulated two independent inputs onto the same population of neurons. We decided to test the effects of the JAK inhibitor AG490 (10 μM), since this inhibitor has been shown to interfere with learning and memory (Chiba et al., 2009b). We found that AG490 had no effect on baseline transmission (100% ± 1% before and 101% ± 1% during AG490 application, n = 13). Next we tested the effects of AG490 on NMDAR-LTP, since this is the most widely studied cellular correlate of learning and memory (Bliss and Collingridge, 1993). However, we found no difference between the level of LTP induced in the control input, in which AG490 was applied immediately after the tetanus, or in the input tetanized in the presence of AG490 (Figure 1A). Thus, the level of LTP obtained 30 min following the tetanus, expressed as a percentage of baseline, was 135% ± 4% and 145% ± 3% (n = 4), respectively. These values were similar to the level of LTP induced in untreated inputs (140% ± 3% of baseline, n = 6; Figure 1C).

Since more recent evidence has suggested that NMDAR-LTD is also involved in some forms of learning and memory (see Collingridge et al., 2010) we next tested AG490 on this form of synaptic plasticity. In all experiments, AG490 completely prevented the induction of NMDAR-LTD induced by low-frequency stimulation (LFS; comprising of 900 stimuli delivered at 1 Hz), though usually a short-term depression remained (Figure 1B). In all cases, the block of NMDAR-LTD was fully reversible since a second, identical period of LFS induced LTD that was similar to that observed under control conditions. Thus, 60 min following the first LFS, delivered in presence of AG490, the responses were 99% ± 4% of baseline and 60 min following the second LFS, delivered after washout of AG490, they were 74% ± 11% of baseline (n = 6). In contrast to the dramatic effect on the induction of NMDAR-LTD, AG490 had no effect on the expression phase of this process. Thus, LFS induced an LTD that was 71% ± 9% and 72% ± 9% of baseline (n = 6), before and following the application of AG490, respectively. Since these experiments were all performed using two inputs, the ability of AG490 to selectively and reversibly block the induction of NMDAR-LTD without affecting baseline transmission or the expression of NMDAR-LTD were all internally controlled.

Next, we explored whether the effects of AG490 were specific for de novo NMDAR-LTD or whether it blocked all forms of LTD. To do this we investigated depotentiation, the reversal of a previously potentiated input. For these experiments we compared, in the two inputs, the level of depotentiation before the application and in the presence of AG490. Under both sets of conditions, LFS reversed LTP to baseline conditions (Figure 1C). For example, in the test input the synaptic response was 137% ± 3% and 93% ± 3% of baseline (n = 6), before and following the LFS.

In conclusion, these experiments have shown that the JAK inhibitor AG490 has a highly specific effect on the induction of NMDAR-LTD.

### Evidence for a Role of Postsynaptic JAK in NMDAR-LTD

**Figure 2:**
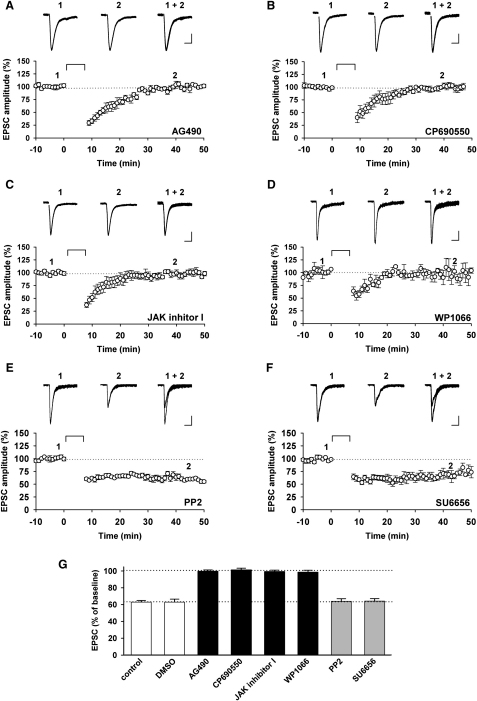
JAK Inhibitors Block NMDAR-LTD (A–F) Representative EPSC traces (top) and pooled data (bottom) of whole-cell patch-clamp recordings illustrating the block of NMDAR-LTD by AG490 (10 μM, n = 6; A), CP690550 (1 μM, n = 5; B), JAK inhibitor I (0.1 μM, n = 6; C), and WP1066 (10 μM, n = 4; D) and the lack of effect of PP2 (10–20 μM, n = 7; E) and SU6656 (10 μM, n = 11; F) on NMDAR-LTD. Calibration bars for all traces: 25 pA/50 ms. (G) Histogram showing the amplitude of NMDAR-LTD, plotted as a percentage of baseline, for the four JAK inhibitors and the two src family PTK inhibitors. Black bars show a significant difference compared to control NMDAR-LTD (with or without DMSO, as appropriate; n = 7 and 28, respectively), and the gray bars show no difference with control.

To establish the locus of action of AG490 we made whole-cell recordings and added the compound to the filling solution (Figure 2). In all neurons loaded with AG490 (10 μM) it was not possible to induce NMDAR-LTD using a pairing protocol (300 pulses, 0.66 Hz, at −40 mV; Figure 2A), whereas in interleaved control experiments NMDAR-LTD was readily induced (Figure 2G). Thus, the responses were 99% ± 2% (n = 6) and 63% ± 4% (n = 7) of baseline, measured at least 30 min after pairing, respectively. These experiments demonstrate that the likely locus of AG490 inhibition is within the postsynaptic neuron. However they do not establish beyond reasonable doubt that the target is JAK since all kinase inhibitors have off-target effects (Bain et al., 2003), due largely to the huge diversity of protein kinases expressed in neurons. The best way to establish the target is to apply a panel of different inhibitors, on the realistic assumption that the off-target effects of the structurally distinct compounds will vary (Peineau et al., 2009). We therefore used three additional JAK inhibitors (CP690550 [1 μM], JAK inhibitor I [0.1 μM], and WP1066 [10 μM]). We also included two src inhibitors (PP2 [10 or 20 μM] or SU6656 [10 μM]) in the study, given that src family PTKs are expressed postsynaptically and regulate neuronal function (Lu et al., 1998; Yu et al., 1997), including insulin-induced LTD (Ahmadian et al., 2004). Similar to the effects of AG490, we found that the other three JAK inhibitors all fully blocked the induction of NMDAR-LTD (101% ± 2% of baseline, n = 5, Figure 2B; 99% ± 2% of baseline, n = 6, Figure 2C; and 99% ± 2% of baseline, n = 4, Figure 2D; respectively). In contrast, neither PP2 nor SU6656 affected the induction of NMDAR-LTD (64% ± 3% of baseline, n = 7, Figure 2E; and 64% ± 3% of baseline, n = 11, Figure 2F; respectively). Apart from blocking the induction of NMDAR-LTD none of the inhibitors affected baseline transmission or other measured properties. The results are summarized in Figure 2G and collectively demonstrate that JAK is required for the induction of NMDAR-LTD.

### JAK2 Knockdown Blocks the Induction of NMDAR-LTD

**Figure 3:**
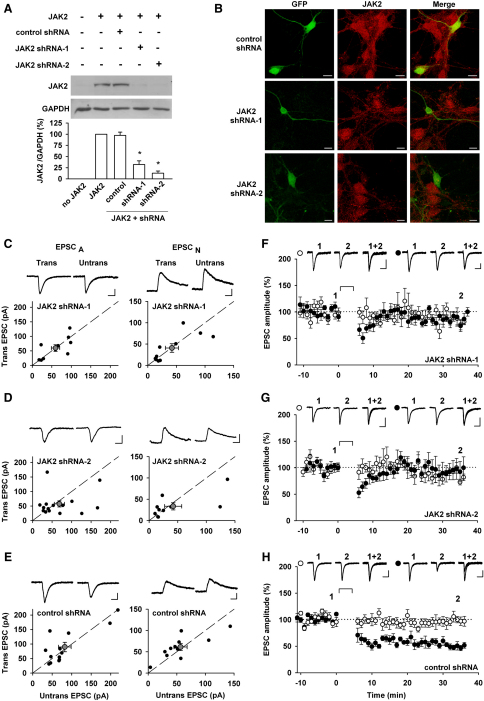
JAK2 Knockdown Blocks the Induction of NMDAR-LTD (A) The efficiency of two JAK2 shRNAs, compared to one control shRNA, was assessed in HEK cells cotransfected with a plasmid coding for JAK2. The values shown are relative to the ratio JAK2/GAPDH obtained from the cells transfected with JAK2 only. ^∗^significantly reduced compared to untransfected cells or the control shRNA. (B) Images of cultured neurons transfected with a plasmid coding for GFP and the control or JAK2 shRNA, as indicated, and labeled with a JAK2 antibody (red). Scale bars: 10 μm. (C–E) AMPAR-mediated EPSCs (EPSC_A_) and NMDAR-mediated EPSCs (EPSC_N_) were recorded in cells transfected (Trans) with a JAK2 shRNA or the control shRNA, as indicated, and nearby untransfected cells (Untrans). Peak amplitudes (EPSC_A_, at −70 mV) or the amplitude measured 60 ms poststimulation (ESPC_N_, at +40 mV) were plotted for each pair (black circles). Gray symbols represent mean ± SEM. Insets show representative traces, which are averages of three successive records. Calibration bars for all traces shown: 40 pA/20 ms. (F–H) The NMDAR-LTD induction protocol failed to induce LTD of EPSC_A_ in cells transfected with the shRNAs for JAK2 (n = 7 and 6 for the first and second JAK2 shRNA tested; F and G) while an input-specific LTD was consistently observed in cells transfected with the control shRNA (n = 8; H). The NMDAR-LTD induction protocol is marked by a horizontal line. Insets show representative traces of the test (black circle) and control (white circle) inputs, before and 25–30 min after LTD induction; each trace is an average of three successive EPSCs. Calibration bars: 50 pA/40 ms.

The available JAK inhibitors do not effectively distinguish between the JAK isoforms. Of the four JAK isoforms present in the body (JAK1, JAK2, JAK3, and TYK2), JAK2 is the most highly expressed in the brain and has been found in the postsynaptic density (PSD) fraction (De-Fraja et al., 1998; Murata et al., 2000). Therefore, to investigate the role of JAK2 in NMDAR-LTD directly, we used constructs coding for two different shRNAs against rat JAK2 or a control shRNA, plus GFP as a transfection marker. The JAK2 shRNAs could effectively knockdown JAK2, as assessed biochemically (shRNA-1: to 32% ± 8% and shRNA-2 to 13% ± 5% of control, n = 5; Figure 3A) and with immunocytochemistry (Figure 3B). We then transfected hippocampal organotypic slices with these constructs to assess their effects on basal synaptic transmission, by comparing AMPAR and NMDAR-mediated EPSCs between pairs of transfected and neighboring untransfected neurons, 48–72 hr after transfection. There was no difference under any condition, showing that knocking down JAK2 has no effect on basal synaptic transmission (Figures 3C–3E). In the next set of experiments we investigated the effects of these constructs on NMDAR-LTD. In all cells examined, NMDAR-LTD was absent in neurons transfected with the JAK2 shRNA constructs (shRNA-1: 88% ± 9% of baseline, n = 7, Figure 3F; shRNA-2: 94% ± 15%, n = 6, Figure 3G). In contrast, NMDAR-LTD was observed in all neurons transfected with the control shRNA (51% ± 5% of baseline, n = 8; Figure 3H), and this was similar to that observed in non-transfected cells (Amici et al., 2009). These experiments further substantiate the pharmacological results identifying a role of JAK in NMDAR-LTD and support the idea that the JAK2 isoform is critically involved in this process.

### JAK2 Is Present in Dendritic Spines and Is Regulated during NMDAR-LTD

**Figure 4:**
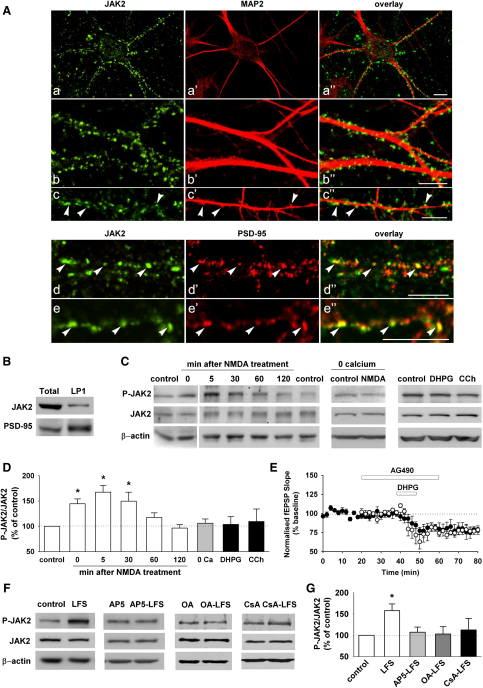
JAK2 Is Present and Regulated at Synapses (A) (a–c″) JAK2 immunoreactivity (green) forms clusters distributed in close apposition with the dendritic arborization of primary hippocampal neurons labeled with MAP2 immunoreactivity (red). Note that a subset of JAK2-immunoreactive clusters are localized at sites of dendritic spine protrusions (arrows in c–c″). (d–e″) Coimmunofluorescence experiments reveals that a subpopulation of JAK2-immunoreactive clusters (green) also expresses PSD-95 immunoreactivity (red) (arrows in d–d″ and e–e″). Scale bars: 10 μm. (B) Biochemical fractionation of hippocampal lysates (Total) shows the presence of JAK2 and the enrichment of PSD-95 in the synaptosomal fraction (LP1). (C) Representative western blots showing JAK2 phosphorylation of Tyr 1007/1008 (P-JAK2, upper), the total amount of JAK2 (middle) and β-actin (lower), in rat hippocampal slices without any treatment (control) or 0 (n = 10), 5 (n = 18), 30 (n = 7), 60 (n = 7), and 120 min (n = 5) after NMDA treatment (20 μM, 3 min); before and after NMDA treatment without calcium (0 Ca); and after DHPG (100 μM, 10 min), and carbachol (CCh, 50 μM, 10 min) treatment. (D) Graph summarizing the level of JAK2 phosphorylation in the different conditions shown in (C), relative to the corresponding control. A significant increase of JAK2 phosphorylation (activation) is observed 0, 5, and 30 min after the NMDA treatment but under no other conditions. (E) Field recording experiments showing DHPG-induced LTD (100 μM, 10 min, white circles, n = 6) is not modified by AG490 (10 μM, 40 min, black circles, n = 5). (F and G) After delivering LFS, the tissue surrounding the stimulated area was analyzed. LFS activates JAK2 in CA1 dendrites (n = 24) but no activation is observed when the slices were treated with AP5 (50 μM, 45 min, n = 10), okadaic acid (OA, 1 μM, 45 min, n = 5) or cyclosporine A (CsA, 50–250 μM, 45 min, n = 5) compared to the corresponding control condition.

We investigated the distribution of JAK2 in cultured hippocampal neurons using confocal microscopy (Figure 4A). JAK2 showed a highly punctate distribution that decorated dendrites, labeled with microtubule-associated protein 2 (MAP2, Figures 4Aa–4Ac″). A high proportion of JAK2 immunostaining was colocalized with PSD-95 (45% ± 3% of PSD-95 positive puncta colocalized with JAK2; 54% ± 3% of JAK2 positive puncta colocalized with PSD-95, Figures 4Ad–4Ae″). We also confirmed, using differential centrifugation, that JAK2 is expressed in the synaptosomal (LP1) fraction (Figure 4B).

If JAK2 is indeed the isoform involved in NMDAR-LTD then it would be expected that its activity would be regulated during the induction of the process. We therefore measured the level of phosphorylation of Tyr 1007/1008, as an indicator of its activity (Feng et al., 1997). In the initial experiments we applied NMDA (20 μM, 3 min), a treatment that induces a chemical form of NMDAR-LTD (Lee et al., 1998). We found that at the three initial time points measured (0, 5, and 30 min after NMDA treatment) the activity of JAK2 in hippocampal slices was significantly increased (145% ± 10%, n = 10; 167% ± 13%, n = 18; 150% ± 18% compared to control, n = 7, respectively; Figures 4C and 4D). However, the activation was transient since there was no significant difference in the level of phosphorylation measured 60 or 120 min later. The activation of JAK was dependent on the presence of Ca^2+^ and was specific for NMDARs, since neither an mGluR agonist (DHPG) nor a muscarinic agonist (carbachol) affected JAK2 phosphorylation (Figures 4C and 4D). Consistent with the lack of effect of DHPG on JAK2 phosphorylation, AG490 had no effect on DHPG-LTD (Figure 4E), a form of LTD induced by the activation of mGluRs (Palmer et al., 1997).

Since there are some differences in the mechanism of LTD induced by bath perfusion of NMDA compared with the LTD induced by synaptic activation of NMDARs (Morishita et al., 2001), we also measured JAK2 phosphorylation in CA1 dendrites following LFS (Figures 4F and 4G). Electrical stimulation also resulted in an increase in JAK2 activity (158% ± 16% compared to nonstimulated slices, n = 24; Figure 4G) and this required the synaptic activation of NMDARs since the increase in phosphorylation was absent in slices treated with AP5 during the LFS (116% ± 14%, n = 10; Figures 4F and 4G). Treatment with inhibitors for the Ser/Thr protein phosphatases PP1 and PP2B also prevented activation of JAK2 during LFS (okadaic acid [1 μM]: 103% ± 17%, n = 9; cyclosporine A [50–250 μM]: 112% ± 27%, n = 5; Figures 4F and 4G).

In summary, the finding that JAK2 is enriched at synapses, colocalizes with PSD-95 and is activated during LTD in an NMDAR, Ca^2+^ and PP1/PP2B dependent manner, suggests that this isoform is involved in NMDAR-LTD.

### STAT3 Is Required for the Induction of NMDAR-LTD

**Figure 5:**
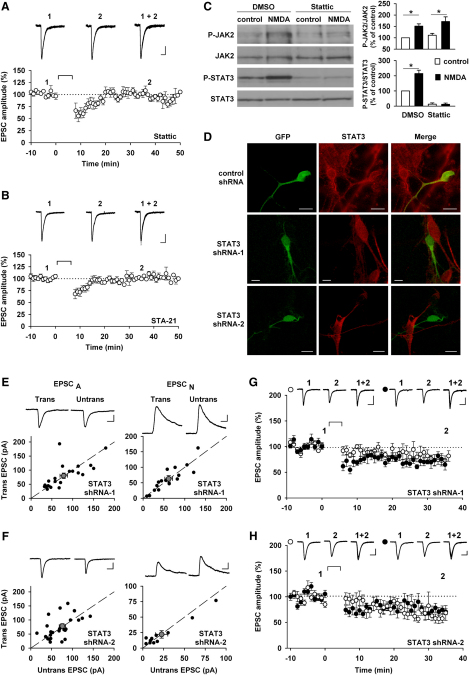
STAT3 Is Required for the Induction of NMDAR-LTD (A and B) Representative EPSC traces (top) and pooled data (bottom) of whole-cell patch-clamp recording illustrating the block of NMDAR-LTD by the STAT3 inhibitors Stattic (50 μM, n = 4; A) and STA-21 (30 μM, n = 7; B). Calibration bars: 40 pA/50 ms. (C) Western blot and bar graphs showing that Stattic (50 μM, 30 min) blocks the activation of STAT3 but not the activation of JAK2 after NMDA treatment (20 μM, 10 min) of cultured hippocampal neurons (n = 4). (D) Two shRNAs against STAT3 and the control shRNA were tested in immunochemistry on neurons in culture transfected with a plasmid coding for GFP and the shRNA. The neurons were then labeled with a STAT3 antibody (red). (E and F) EPSC_A_ and EPSC_N_ (as in Figures 3C–3E) were recorded from neurons transfected with 2 different STAT3 shRNA and nearby non transfected neurons. Calibration bars: 40 pA/20 ms. (G and H) Representative traces and pooled data, presented as in Figures 3F–3H, showing that homosynaptic NMDAR-LTD was not observed in cells transfected with the first (G) and second (H) shRNA against STAT3 (n = 8 for both). The experiments with the control shRNA were interleaved (Figure 3H). Calibration bars: 50 pA/40 ms

The next question we wished to address is what the downstream effector of JAK2 is in NMDAR-LTD. JAK2 is known to signal via the PI3K/Akt pathway and the ras/MAPK pathway (Lanning and Carter-Su, 2006; Zhu et al., 2001). However, inhibitors of these pathways do not affect NMDAR-LTD, under our experimental conditions (Peineau et al., 2009). Another possibility is via STATs. The JAK/STAT pathway is a major signaling pathway involved in many nonneuronal processes where JAK activation leads to phosphorylation of STATs, which results in their activation and translocation to the nucleus. We focused on STAT3, since this isoform is present in the hippocampus and PSD (Cattaneo et al., 1999; De-Fraja et al., 1998; Murata et al., 2000). Therefore, we tested the effects of two compounds that inhibit the activation of STAT3: Stattic (50 μM) and STA-21 (30 μM). We found that both STAT3 inhibitors prevented the induction of NMDAR-LTD (99% ± 2% of baseline, n = 4, Figure 5A; and 96% ± 4% of baseline, n = 7, Figure 5B; respectively), with a similar time-course as the JAK inhibitors.

These data are consistent with a scheme in which, during NMDAR-LTD, activation of JAK2 leads to activation of STAT3. In which case, inhibition of STAT3 would not be expected to affect the activation of JAK2 (Beales and Ogunwobi, 2009; Schust et al., 2006). To test whether this was indeed the case, we treated cultured hippocampal neurons with Stattic and found that this completely prevented the activation of STAT3 without affecting the activation of JAK2 in response to the stimulation of NMDARs (Figure 5C). This treatment also reduced basal levels of STAT3 activity suggesting that there is a degree of constitutive activation of STAT3.

To substantiate the involvement of STAT3 in NMDAR-LTD, we used two different shRNAs against STAT3, which efficiently knocked down the target protein in hippocampal cultured neurons as assessed with immunocytochemistry (Figure 5D). As with JAK2 shRNAs, the knockdown of STAT3 had no effect on basal synaptic transmission, as assessed by comparing AMPAR and NMDAR-mediated EPSCs between pairs of transfected and neighboring untransfected neurons, 48–72 hr after transfection in organotypic slices (Figures 5E and 5F). However, no NMDAR-LTD could be observed in the cells transfected with the shRNAs (Figures 5G and 5H), whereas NMDAR-LTD was reliably induced in interleaved experiments in neurons transfected with control shRNA (Figure 3H). With both shRNAs against STAT3 there was a small decrease in the synaptic response following the LTD stimulus protocol but this was similar for both the test and control inputs, and significantly smaller than for control LTD. When all these data are considered together it strongly suggests that STAT3 is the isoform involved in NMDAR-LTD.

### STAT3 Is Activated and Translocated to the Nucleus during NMDAR-LTD

**Figure 6:**
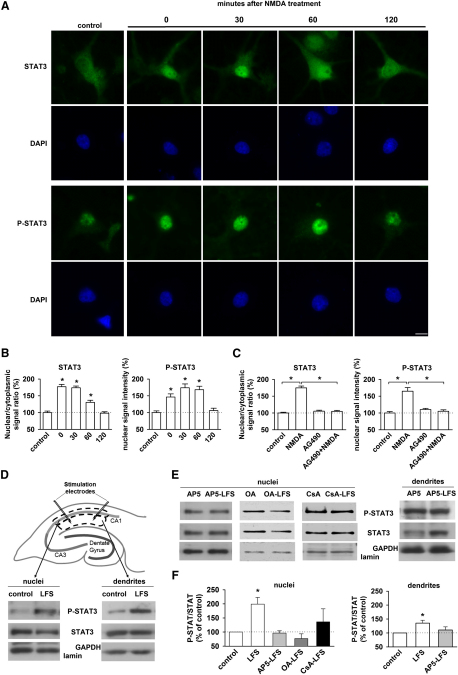
STAT3 Is Activated and Translocated to the Nucleus during NMDAR-LTD (A) STAT3 and P-STAT3 (Tyr 705) immunoreactivity in the cytoplasm and nuclei of hippocampal neurons in culture. NMDA treatment (20 μM, 10 min) induces an increase of both STAT3 and P-STAT3 immunoreactivity in neuronal nuclei, 0, 30, and 60 min after NMDA treatment. Scale bar: 10 μm. (B) The graphs show that the nuclear/cytoplasmic STAT3 signal ratio and the P-STAT3 signal intensity are significantly increased at these times, compared with control values. (C) AG490 (10 μM) pretreatment prevents the NMDA-induced increase of nuclear STAT3 and P-STAT3 immunoreactivity. (D) After delivering LFS, the stratum radiatum surrounding the stimulating electrodes (dendrites) or the stratum pyramidale (CA1 cells bodies) close to the stimulated area were microdissected. The nuclei from the cell bodies were isolated by centrifugation. The blots of P-STAT3 and STAT3 show that the phosphorylation of STAT3 was increased in both compartments. Lamin and GAPDH were used as a blotting control for the nuclei and dendrites, respectively. (E) The blots show that no activation of STAT3 is observed when the slices are treated with AP5 (50 μM, 45 min), okadaic acid and cyclosporin A (as in Figures 4F and 4G) prior and during LFS. (F) Graphs summarizing the data obtained in (D) (n = 14) and (E) (n = 10 for AP5 and n = 5 for okadaic acid and cyclosporin A).

Since, when activated, STAT3 translocates to the nucleus, we wanted to see if this activation and translocation also occurs during NMDAR-LTD. In cultured hippocampal neurons under control conditions, STAT3 immunoreactivity was fairly evenly distributed throughout the neuron, including the nucleus (Figure 6A). NMDA treatment (20 μM, 10 min) resulted in nuclear translocation and activation of STAT3 (Figure 6A). Maximal nuclear accumulation was observed immediately following NMDAR stimulation and the effect persisted for between 1 and 2 hr (Figures 6A and 6B). There was a corresponding activation of nuclear STAT3, as assessed by the phosphorylation of Tyr 705 (P-STAT3), which also lasted for between 1 and 2 hr (Figures 6A and 6B). Consistent with the activation of STAT3 being mediated by JAK2, treatment of cultures with AG490 prevented both the translocation of STAT3 and activation of nuclear STAT3 (Figure 6C).

To investigate whether STAT3 is also activated by LFS in hippocampal slices, we analyzed the levels of STAT3 and P-STAT3 in the CA1 region of hippocampal slices by western blotting. For these experiments, we microdissected both stratum radiatum, which is enriched in CA1 dendrites, and stratum pyramidale, which is correspondingly enriched in CA1 cell soma (Figure 6D). We prepared a nuclear fraction from the microdissected cell soma preparation and examined the expression of P-STAT3 relative to total STAT3. LFS resulted in a pronounced activation of nuclear STAT3 (199% ± 23%, n = 14, Figures 6D and 6F), which was absent if LFS was delivered in the presence of AP5 (94% ± 8%, n = 10), okadaic acid (87% ± 17%, n = 5) or cyclosporine A (136% ± 46%, n = 5; Figures 6E and 6F). Interestingly, LFS also resulted in activation of dendritic STAT3 (135% ± 10%, n = 14; Figures 6D and 6F) and this effect was also dependent on the synaptic activation of NMDARs (110% ± 11% in presence of AP5, n = 10; Figures 6E and 6F). These results are consistent with the immunocytochemistry (Figures 6A and 6B) in cultured neurons and extend them by showing the dependence of nuclear STAT3 activation on the PP1/PP2B protein phosphatase cascade. Significantly, these results show that the synaptic activation of NMDARs can lead to the activation of STAT3 in dendrites.

### The Translocation of STAT3 to the Nucleus Is Not Required for NMDAR-LTD

Once activated, cytoplasmic STATs are translocated to the nucleus where they bind to DNA specific sequences within the promoter region to control gene expression. There is evidence that rapid transcription may be involved in LTD (Kauderer and Kandel, 2000). Therefore, to investigate whether the rapid effect of inhibition of STAT3 on NMDAR-LTD was due to interference with gene transcription we performed a variety of different experiments.

**Figure 7:**
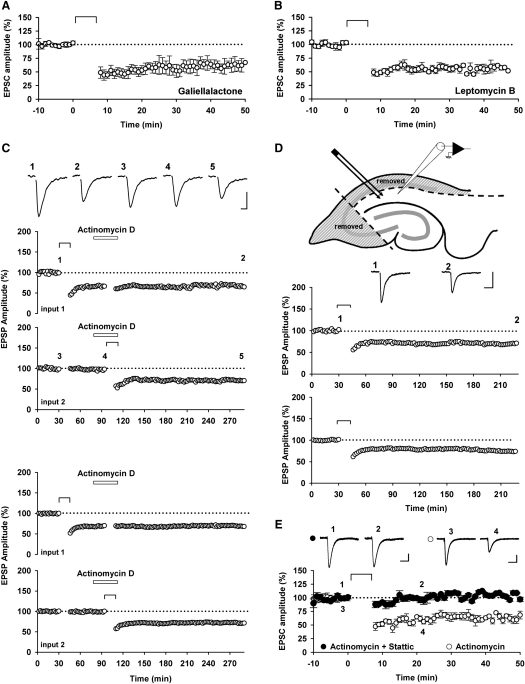
Translocation of STAT3 to the Nucleus Is Not Necessary for NMDAR-LTD (A) Pooled data of EPSC amplitudes in whole-cell patch-clamp recording showing that NMDAR-LTD can be induced in presence of galiellalactone (50 μM; n = 5), a STAT3 inhibitor which block its binding to DNA. (B) NMDAR-LTD can also be induced in presence of leptomycin B (50 nM; n = 6), a blocker of nuclear translocation. (C) Experiments performed with field-recording, as in Figure 1, show that actinomycin D (25 μM) does not block pre-established NMDAR-LTD or the induction of NMDAR-LTD even 3 hr after induction (n = 4). Calibration bars: 0.5 mV/20 ms. (D) Experiments performed with field-recording in slices where the cell bodies have been removed, as shown on the diagram, showing that the nucleus is not required for the induction of NMDAR-LTD (n = 5). Calibration bars: 0.5 mV/40 ms. (E) Stattic (50 μM) can still block the induction of NMDAR-LTD even in presence of actinomycin D (25 μM), a transcription inhibitor (n = 5 for both conditions). Calibration bars: 25 pA / 40 ms.

We first tested galiellalactone, a STAT3 inhibitor that blocks STAT3 binding to DNA without affecting STAT3 activation. In all neurons loaded with galiellalactone (50 μM) NMDAR-LTD was readily induced (58% ± 8% of baseline, n = 5; Figure 7A). To further explore whether nuclear signaling is required for NMDAR-LTD, we used a nuclear export inhibitor (leptomycin B, 50 nM) and this also failed to inhibit NMDAR-LTD (57% ± 3% of baseline, n = 6; Figure 7B). To investigate transcription more generally, we tested the effects of actinomycin D (25 μM). In field recordings we followed NMDAR-LTD for 3 hr after induction and observed no difference between the level of LTD in the control and test inputs (69% ± 3% and 71% ± 2% of baseline, n = 4, respectively; Figure 7C). We also performed experiments in slices from which the cell body region of the slice had been completely removed. Once again, NMDAR-LTD that lasted at least 3 hr could be readily observed (76% ± 3% of baseline, n = 5, Figure 7D). These data collectively suggest that NMDAR-LTD can be readily induced and expressed for at least 3 hr, without the need for gene transcription and that the effects of inhibition of STAT3 are independent of an action within the nucleus. As a final test of this, we blocked transcription using actinomycin D (25 μM) in the patch pipette and tested the effects of Stattic under these conditions. In all neurons tested, NMDAR-LTD was readily induced in the presence of actinomycin D (63% ± 3% of baseline, n = 5; Figure 7E) but was fully blocked by the additional inclusion of Stattic (50 μM) in the patch solution (98% ± 2% of baseline, n = 5; Figure 7E).

In summary, activation of STAT3, but not its binding to DNA, is required for the induction and early expression of NMDAR-LTD.

## Discussion

**Figure 8:**
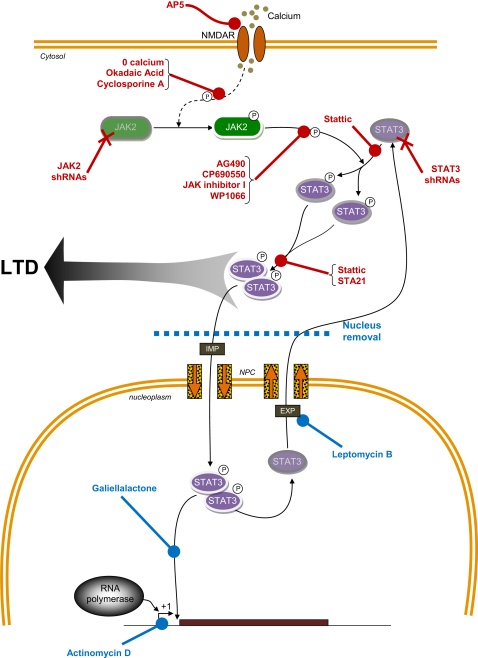
Schematic Representation of the Role of the JAK/STAT Pathway in NMDAR-LTD Stimulation of NMDARs leads to activation of JAK2 (phosphorylation) via a pathway involving Ca^2+^, PP2B and PP1. Activated JAK2 leads to activation of STAT3, which then translocates to the nucleus. STAT3 also has cytoplasmic actions, which are required for NMDAR-LTD. The 14 treatments in red all inhibit NMDAR-LTD, whereas the 4 treatments in blue do not. IMP: importin; NPC: nuclear pore complex; EXP: exportin.

In the present study, we have shown that the JAK/STAT pathway is engaged by the synaptic activation of NMDARs and that it is required for the induction of NMDAR-LTD. The involvement of the JAK/STAT pathway is specific for this form of LTD since it was not involved in either depotentiation or mGluR-LTD and is also not involved in LTP. While we cannot exclude a role of the JAK/STAT pathway in other forms of synaptic plasticity, for example in other regions of the CNS or under different experimental conditions, these findings further support the notion that a set of distinct molecules are associated with the different major forms of synaptic plasticity in the CNS (i.e., NMDAR-dependent and NMDAR-independent LTP and LTD). A scheme that summarizes our observations is presented in Figure 8.

### Mechanisms Involved in NMDAR-LTD

NMDAR-LTD has been the subject of considerable recent interest with the increasing realization that this process is involved in learning and memory and various pathological processes. However, the understanding of its molecular mechanism is incomplete. The first step involves Ca^2+^ entry via NMDARs (Cummings et al., 1996) and Ca^2+^ release from intracellular stores (Daw et al., 2002; Reyes and Stanton, 1996). This intracellular calcium increase leads to the activation of several Ca^2+^-dependent proteins, including calmodulin (Mulkey et al., 1993), hippocalcin (Palmer et al., 2005), and protein interacting with C-kinase 1 (PICK1) (Terashima et al., 2008) and to the activation of the caspase-3 signaling pathway through mitochondrial stimulation (Li et al., 2010). The multiple calcium sensors then interact with several downstream effectors involved in AMPAR trafficking, including ABP/GRIP (Chung et al., 2000), AP2 (Lee et al., 2002; Palmer et al., 2005), the Arp2/3 complex (Nakamura et al., 2011; Rocca et al., 2008), PSD-95 and AKAP (Bhattacharyya et al., 2009; Kim et al., 2007), Rab5a (Brown et al., 2005), as well as RalBP1 (Han et al., 2009). These processes are all dependent on, and regulated by, protein phosphorylation.

In this regard, there is strong evidence for the involvement of a Ser/Thr protein phosphatase cascade involving protein phosphatase 2B (calcineurin) and protein phosphatase 1 (Mulkey et al., 1993, 1994) and the dephosphorylation of Ser845 of GluA1 (Lee et al., 1998). In addition, there is also evidence for the involvement of the Ser/Thr kinase, glycogen synthase kinase-3 β (GSK-3β) (Peineau et al., 2007, 2009) and inhibition of the activity of protein kinase M zeta (PKMζ) (Hrabetova and Sacktor, 1996). A role for tyrosine phosphorylation also appears to be important (Ahmadian et al., 2004; Hayashi and Huganir, 2004) though the mechanism of its involvement is not yet understood.

Clearly, a fuller understanding of NMDAR-LTD is important given its relevance to both learning and memory and various neurological diseases. However, before this can be achieved the major signaling pathways involved need to be identified.

### A Role for JAK2 in NMDAR-LTD

Our conclusion that a member of the Janus kinases, JAK2, is involved in NMDAR-LTD is based on several lines of complementary evidence.

First, we identified a role of JAK pharmacologically. The extracellular recording experiments showed that the role of JAK is specific for the induction of this one form of synaptic plasticity, since baseline transmission, pre-established NMDAR-LTD, depotentiation, mGluR-LTD and LTP were all unaffected by a concentration of a JAK inhibitor that fully prevented the induction of NMDAR-LTD.

Second, by loading inhibitors into neurons via the patch pipette we were able to demonstrate that the site of action was likely to be postsynaptic, which is consistent with the known locus of induction of NMDAR-LTD as well as with the postsynaptic localization of both JAK2 and STAT3. In addition, we compared a panel of different inhibitors (Figure 8), which is important due to off-target effects of all kinases inhibitors (Bain et al., 2003; Peineau et al., 2009). The likelihood of four structurally distinct compounds all having the same off-target effect that explains the block of NMDAR-LTD is remote indeed. Consistent with the extracellular experiments, there was no effect on baseline transmission, which would have been observed as an alteration in EPSC amplitude upon obtaining the whole-cell recording. No alterations in other neuronal properties were observed. Collectively, therefore, these results demonstrate a highly specific role for JAK in NMDAR-LTD.

Third, we found that knockdown of the JAK2 isoform also resulted in abolition of NMDAR-LTD. Given JAKs are important for cell survival we were concerned that these knockdown experiments would not be feasible. However, we found that it was possible to perform experiments within 48–72 hr of transfection at a time when neurons were healthy and both AMPAR- and NMDAR-mediated synaptic transmission were unaffected. The elimination of NMDAR-LTD was not a consequence of transfection since the control shRNA had no effect on NMDAR-LTD.

Fourth, we found that the JAK2 isoform was heavily expressed at synapses, thereby positioning the enzyme in the right location to be involved in synaptic plasticity. We have focused on JAK2, since this isoform is the most highly expressed in the CNS. In particular, whereas JAK2 is expressed in the PSD, JAK1, JAK3, and TYK2 have not been detected in this structure (Murata et al., 2000). Although it seems unlikely, therefore, that other JAK isoforms are also involved in NMDAR-LTD, a role of one or more of these isoforms in other synaptic processes cannot be discounted.

Lastly, we found that the activity of JAK2 was increased during NMDAR-LTD. Again, this effect was specifically related to the synaptic activation of NMDARs and the entry of calcium. The activation of JAK2 also depended on the phosphatases PP1 and PP2B, which are critically involved in NMDAR-LTD (Mulkey et al., 1993). These data suggest that JAK2 is downstream of the Ser/Thr protein phosphatase cascade, but further work will be required to establish the full details of its activation pathway. Proteins of potential interest in this respect are GSK3β, possibly via inhibition of Src homology-2 domain-containing phosphatase (SHP) 2 (Kai et al., 2010; Tsai et al., 2009) and/or proline-rich tyrosine kinase 2, PYK2, which has been found to be involved in LTD (Hsin et al., 2010) and which, in nonneuronal systems, has been shown to associate with and activate JAK (Frank et al., 2002; Takaoka et al., 1999).

### A Role for STAT3 in NMDAR-LTD

Having established a role for JAK2 in NMDAR-LTD we next wished to identify its downstream effector in this process. We discounted the class 1A PI3K and MAPK pathways since inhibition of these cascades does not affect NMDAR-LTD at these synapses under the conditions of our experiments (Peineau et al., 2009). Instead, we focused on the STATs since these are well established targets of JAKs in a wide variety of homeostatic functions. There are many STAT isoforms so we focused our attention on STAT3, since this is a common partner of JAK2 and is also expressed at the PSD (Murata et al., 2000). Again, we obtained complementary evidence for a role of STAT3 in NMDAR-LTD.

First, we found that two structurally unrelated inhibitors of STAT, with selectivity toward STAT3, were able to block NMDAR-LTD (Figure 8). Surprisingly, NMDAR-LTD was blocked fairly rapidly, with a time course similar to that seen with the JAK2 inhibitors. We confirmed that Stattic was able to inhibit the activation of STAT3 without affecting the activation of JAK2, which is consistent with a specific action downstream of JAK2.

Second, two different STAT3 shRNAs also blocked NMDAR-LTD reinforcing the role of this isoform in NMDAR-LTD. Since STAT3 is a transcription factor involved in cell survival, using a knockdown approach to investigate its physiological role has limitations. The experiments were performed 2–3 days after transfection on CA1 cells that appeared healthy by visual inspection. We found that both AMPAR and NMDAR-mediated synaptic transmission was unaffected by knockdown of STAT3. However, the LFS induction protocol resulted in a small rundown in synaptic transmission in both inputs. Further experiments will be required to establish the origin of this effect. With respect to NMDAR-LTD, however, there was no difference between the control and test inputs. These data fully support the conclusions from the pharmacological experiments that activation of STAT3 is required for NMDAR-LTD.

Third, we observed a translocation of STAT3 from the cytoplasm to the nucleus upon NMDAR stimulation in cultured hippocampal neurons. This effect was associated with an increase in activity of nuclear STAT3, as assessed by its phosphorylation status. The activation of nuclear STAT3 was dependent on JAK2 activation and they both had a similar time course, which suggests that the kinetics of the pathway is determined primarily by the activation status of JAK2.

Fourth, we found that nuclear STAT3 was also activated by the synaptic activation of NMDARs in hippocampal slices and, similarly to JAK2, this effect also required PP1 and PP2B. STAT3 activation was, unsurprisingly, most prominent in the nucleus but there was also a significant activation of cytoplasmic STAT3 in the dendritic fraction. While this is not unexpected, since STATs are phosphorylated in the cytoplasm before they are translocated into the nucleus, it could enable STAT3 to have an additional signaling function outside of the nucleus.

Finally, we established that STAT3 does not play a role in NMDAR-LTD via its role in transcription, by using a variety of different approaches (Figure 8). Thus, inhibiting the binding of STAT3 to DNA, preventing nuclear export, blocking transcription, and removing the cell body region of the slice, which contains the vast majority of neuronal soma, were all unable to block the induction of NMDAR-LTD. Our finding that NMDAR-LTD is independent of transcription differs from a previous report (Kauderer and Kandel, 2000) for reasons that are unclear. Of course, we cannot discount a role of transcription at times beyond the 3 hr that we have investigated here. Indeed, a plausible role for the increase in nuclear STAT3 activity that we have observed may be in the regulation of proteins that are required for later phases of the NMDAR-LTD process.

Our findings strongly suggest that STAT3 has nonnuclear actions that are required for NMDAR-LTD. Unfortunately, little is known concerning the role of STATs on targets other than DNA. Recent evidence has implicated the regulation of microtubules in NMDAR-LTD (Kapitein et al., 2011). Interestingly, it has been shown that STAT3 can directly interact with proteins associated with microtubules, such as stathmin and SCG10-like protein (SCLIP), and regulate their polymerization (Gao and Bromberg, 2006; Ng et al., 2006, 2010). One possibility then is that STAT3 could regulate the stabilization of microtubules, a mechanism that is believed to be rapid, dynamic and reversible (Gao and Bromberg, 2006).

### Clinical Implications

The role of JAKs in oncogenesis and pathologies of the immune system make these kinases attractive potential therapeutic targets. In particular, JAK2 mutations underlie the myeloproliferative disorders: polycythemia vera, essential thrombocytosis, and primary myelofibrosis (Delhommeau et al., 2010). Since JAK2 is overactivated in these pathologies, a specific JAK inhibitor has potential utility in the treatment of these diseases and several clinical trials for JAK2 inhibitors are underway (Quintás-Cardama et al., 2011). However, the effect of available JAK2 inhibitors on the other JAK isoforms and the inhibition of the central role JAKs play as downstream effectors of cytokine receptors have been major issues so far (Pesu et al., 2008; Wilks, 2008). The JAK2 inhibitor AG490 has also been shown to affect spatial learning and memory (Chiba et al., 2009b). It was suggested that this impairment was due to the downregulation of the enzyme choline acetyltransferase and to the desensitization of the M1-type muscarinic acetylcholine receptor (Chiba et al., 2009b). We now show that inhibiting JAK2 results in blockade of a specific form of synaptic plasticity, NMDAR-LTD.

## Experimental Procedures

A complete description of experimental procedures is available online in the supplemental information.

### Inhibitors and shRNA Plasmids

A complete list of the inhibitors used is available in the supplemental information. Organotypic slices were transfected using biolistic transfection with HuSH shRNA constructs in pGFP-V-RS vector (Origene Technologies, Rockville, MD, USA). The sequences of the 29-mer shRNAs against rat JAK2 were GCCATCAGCAAACTAAAGAAGGCAGGAAA for the shRNA-1 and CAGCCTGTTTACTCCAGATTATGAACTGC for the shRNA-2. The sequences for the shRNAs against rat STAT3 were ACTGGATAACTTCATTAGCAGAATCTCAA for the shRNA-1 and TTCTTCACTAAGCCTCCGATTGGAACCTG for the shRNA-2. The sequence of the control, noneffective, shRNA used was GCACTACCAGAGCTAACTCAGATAGTACT.

### Preparation of Slices

The experiments on acute slices were performed on 400 μm thick parasagittal hippocampal slices obtained from juvenile (13- to 17-day-old) Wistar rats, as described previously (Peineau et al., 2007). Hippocampal organotypic slices were prepared from 8-day-old Wistar rats, as described previously (Bortolotto et al., 2011; Jo et al., 2010). Procedures involving animals and their care were conducted in conformity with the institutional guidelines that are in compliance with national (UK animals (Scientific Procedures) Act 1986 and D.L.n.116, G.U., Suppl. 40, 1992) and international laws and policies (EEC Council Directive 86/609, OJ L 358, 1, 12 December 1987; Guide for the Care and Use of Laboratory Animals, U.S. National Research Council, 1996).

### Electrophysiology

In all electrophysiology experiments, the CA3 region was removed. Extracellular and whole-cell experiments were performed as reported previously (Peineau et al., 2007).

Whole-cell voltage-clamp recordings on organotypic slices were made from transfected CA1 pyramidal cells at 6–11 days in vitro and were performed blind with respect to the transfected plasmid. Two stimulating electrodes (test and control input) were placed in the Schaffer collateral-commissural pathway and stimulated at 0.05 Hz to record AMPAR EPSCs (V_h_ = −70 mV). To measure NMDAR EPSCs, neurons were held at +40 mV and the EPSC amplitude was measured 60 ms following the stimulus. NMDAR-LTD was induced using a pairing protocol (1 Hz for 6 min, V_h_ = −40 mV). Access resistance was monitored constantly and neurons were discarded if this varied by more than 20% during the recording period. Data were stored and analyzed using the WinLTP Program (Anderson and Collingridge, 2007) and are presented as mean ± SEM.

### Biochemistry

For chemically induced LTD, whole hippocampal slices (without CA3) were treated with either 20 μM NMDA for 3 min, 100 μM DHPG for 10 min or 50 μM carbachol for 10 min. For LFS-induced LTD, hippocampal slices were stimulated (900 stimulations at 1 Hz) with two electrodes placed in the Schaffer collateral-commissural fibers. The stratum radiatum surrounding the stimulating electrodes, enriched in CA1 dendrites, and the stratum pyramidale, enriched in CA1 cell bodies, were then microdissected within the next 10 min and washed in a cold buffer. The nuclei were isolated by centrifugation from the stratum pyramidale portion. The samples were then lysed and western blotting was performed. The antibodies used were: rabbit anti-phospho-JAK2 (Tyr 1007-1008, 09-275, Millipore, Billerica, MA, 1:500), rabbit anti-JAK2 (3230, Cell Signaling Technology, Danvers, MA, 1:500), mouse anti-phospho-STAT3 (Tyr 705, sc-8059, Santa Cruz Biotechnology, Santa Cruz, CA, 1:100), mouse anti-STAT3 (sc-8019, Santa Cruz Biotechnology, 1:100), mouse anti-β-actin (ab6276, Abcam, Cambridge, MA, 1:10,000), rabbit anti-GAPDH (2118, Cell Signaling Technology, 1:10,000), rabbit anti-lamin (2032, Cell Signaling Technology, 1:200) and mouse or rabbit secondary antibodies (Millipore, 1:10,000).

### Hippocampal Cell Cultures

For STAT3 translocation to the nucleus, hippocampi were dissected from 18-day-old embryo Sprague-Dawley rat brains. Mature cultured neurons (day in vitro; DIV12) were treated with 20 μM D-serine as control or 20 μM D-serine + 20 μM NMDA for 10 min at 37°C and fixed at different time after the treatment. Some neurons were pretreated with 10 μM AG490 for 30 min and fixed immediately after the NMDA treatment.

To test the efficiency of the shRNAs and for the experiments with the inhibitor Stattic, hippocampi were dissected and dissociated and cultured from 2-day-old Wistar rats.

Transfection of the cells with the shRNAs was performed at DIV 4–6 using lipofectamine according to the manufacturer's protocol and the cells were fixed 2–3 days later.

Pharmacological treatment with D-Serine and NMDA was performed as described above, at DIV 4–8, on cells incubated with either vehicle control DMSO or Stattic (50 μM) for 20–30 min. Cells were then washed and lysed in a standard lysis buffer.

### Immunocytochemistry

Neurons were fixed with paraformaldehyde 4% or methanol and incubated in a donkey serum blocking buffer before labeling them with JAK2 (sc-278; Santa Cruz Biotechnology; 1:50 or ab39636, Abcam; 1:200), MAP2 (ab11268; Abcam; 1:1,000), PSD-95 (05-494; Upstate Biotechnology, Billerica, MA; 1:200), STAT3 (124H6; Cell Signaling Technology; 1:400), or phospho-STAT3 (9131; Cell Signaling Technology; 1:100). The coverslips were then mounted with Fluoromount for microscopic observations. See supplemental experimental procedures for details.

### HEK Cells and Transfection

HEK293 cells were transfected with a pcDNA3-rJAK2(FL)-HA plasmid and the different shRNAs, using an Amaxa Nucleofector Kit V according to the manufacturer's instructions. The cells were lysed in a standard lysis buffer 72 hr after transfection, and the levels of JAK2 and GAPDH were analyzed by western blot.

### Statistics

Two-tailed paired or unpaired Student's t tests or one-way ANOVA were carried out as appropriate, with a significance level set at p < 0.05 (and indicated in the figures by an asterisk).

## Supplemental Information

Document S1. Supplemental Experimental Procedures
