# Supplementary material for: The JAK/STAT Pathway Is Involved in Synaptic Plasticity
Source: Neuron. 2012 Jan 26;73(2):374–90. doi: 10.1016/j.neuron.2011.11.024 (PMC3268861; doi:10.1016/j.neuron.2011.11.024)
Supplement: Document S1. Supplemental Experimental Procedures [file mmc1.pdf]

Article

The JAK/STAT Pathway Is Involved in Synaptic Plasticity

Céline S. Nicolas, Stéphane Peineau, Mascia Amici, Zsolt Csaba, Assia Fafouri, Charlotte Javalet, Valerie J. Collett, Lars Hildebrandt, Gillian Seaton, Sun-Lim Choi, Su-Eon Sim, Clarrisa Bradley, Kyungmin Lee, Min Zhuo, Bong-Kiun Kaang, Pierre Gressens, Pascal Dournaud, Stephen M. Fitzjohn, Zuner A. Bortolotto, Kwangwook Cho, and Graham L. Collingridge

SUPPLEMENTAL EXPERIMENTAL PROCEDURES

Inhibitors and shRNA plasmids

The following compounds were used: DMSO (dimethyl sulfoxide, from Sigma-Aldrich, St. Louis, MO), AG490 ((E)-N-benzyl-2-cyano-3-(3,4-dihydroxyphenyl)acrylamide a-cyano-(3,4-dihydroxy)-N-benzylcinnamide), JAK inhibitor I (2-(1,1-dimethylethyl)-9-fluoro-3,6-dihydro-7H-benz[h]-imidaz[4,5-f]isoquinolin-7-one), SU6656 (2,3-dihydro-N,N-dimethyl-2-oxo-3-[(4,5,6,7-tetrahydro-1H-indol-2-yl)methylene]-1H-indole-5-sulfonamide) (from Calbiochem, Darmstadt, D), actinomycin D (2-Amino-(*N*,*N*)-1-*bis*(hexadecahydro-6,13-diisopropyl-2,5,9-trimethyl-1,4,7,11,14-pentaoxo-1*H*-pyrrolo[2,1]-[1,4,7,10,13] oxatetraazacyclohexadecin-10-yl)-4,6-dimethyl-3-oxo-3*H*-phenoxazine-1,9-dicarboxamide), AP-5 ((2R)-amino-5-phosphonovaleric acid), cyclosporine A ({R-[R*,R*-(E)]}-cyclic-(L-alanyl-D-alanyl-N-methyl-L-leucyl-N-methyl-L-leucyl-N-methyl-L-valyl-3-hydroxy-N,4-dimethyl-L-2-amino-6-octenoyl-L- a-amino-butyric-N-methyl-glycinyl-N-methyl-L-leucyl-L-valyl-N-methyl-leucyl) , leptomycin B ((2*E*,5*S*,6*R*,7*S*,9*R*,10*E*,12*E*,15*R*,16*Z*,18*E*)-19-[(2*S*,3*S*)-3,6-Dihydro-3-methyl-6-oxo-2*H*-pyran-2-yl]-17-ethyl-6-hydroxy-3,5,7,9,11,15-hexamethyl-8-oxo-2,10,12,16,18-nonadecapentaenoic acid), okadaic acid (9,10-Deepithio-9,10-didehydroacanthifolicin), PP2 (3-(4-chlorophenyl)1-(1,1-dimethylethyl)-1H-pyrazolo[3,4-d]pyrimidin-4-amine), Stattic (6-Nitrobenzo[b]thiophene 1,1-dioxide) (from Tocris Cookson, Avonmouth, UK), CP690550 (3-{(3R,4R)-4-methyl-3-[methyl-(7H-pyrrolo[2,3-d]pyrimidin-4-yl)-amino]-piperidin-1-yl}-3-oxo-propionitrile) (from Axon Medchem, Groningen, NL), WP1066 (from Sigma-Aldrich, St. Louis, MO, USA), STA-21 ((S)-Ochromycinone deoxytetrangomycin) (from Santa-cruz, CA, USA) and galiellalactone (form Tebu-bio, Smithfield, Australia) Organotypic slices were transfected using biolistic transfection with HuSH shRNA constructs in pGFP-V-RS vector (Origene Technologies, Rockville, MD, USA). The

control shRNA used was a scrambled negative control non-effective shRNA: GCACTACCAGAGCTAACTCAGATAGTACT. The sequences of the 29mer shRNAs against rat JAK2 were GCCATCAGCAAACTAAAGAAGGCAGGAAA for the shRNA-1 and CAGCCTGTTTACTCCAGATTATGAACTGC for the shRNA-2. The sequences for the shRNAs against rat STAT3 were ACTGGATAACTTCATTAGCAGAATCTCAA for the shRNA-1 and TTCTTCACTAAGCCTCCGATTGGAACCTG for the shRNA-2.

Preparation of slices

The experiments on acute slices were performed on 400 µm thick parasagittal hippocampal slices obtained from juvenile (13 - 17 day old) Wistar rats. Hippocampal organotypic slices were prepared from 8 day old Wistar rats, as described previously (Bortolotto et al., 2011; Jo et al., 2010). Procedures involving animals and their care were conducted in conformity with the institutional guidelines that are in compliance with national (UK animals (Scientific Procedures) Act 1986 and D.L.n.116, G.U., Suppl. 40, 1992) and international laws and policies (EEC Council Directive 86/609, OJ L 358, 1, 12 December 1987; Guide for the Care and Use of Laboratory Animals, U.S. National Research Council, 1996).

Electrophysiology

In all electrophysiology experiments, the CA3 region was removed. For extracellular recording, slices were maintained in a medium comprising (mM:) NaCl, 124; KCl, 3; $NaHCO_3$, 26; NaH_2PO_4, 1.25; $CaCl_2$, 2; $MgSO_4$, 1; D-glucose, 10; picrotoxin, 50; bubbled with O_2:CO_2: 95:5%. The Schaffer collateral-commissural pathway was stimulated at 30 s intervals for each input (stimuli were delivered every 15 s to alternate inputs). Individual responses were displayed and the average field EPSP amplitude of four successive responses were plotted on-line using WinLTP software (Anderson and Collingridge, 2007) and normalized to baseline. The NMDAR-LTD was induced by delivering 900 shocks at 1 Hz (LFS) and LTP was induced by delivering 100 shocks at 100 Hz, both at test intensity.

For whole-cell experiments, the slices were perfused with artificial cerebrospinal fluid (ACSF) which comprised (mM): NaCl, 124; KCl, 3; $NaHCO_3$, 26; NaH_2PO_4, 1.25; $CaCl_2$, 2; $MgSO_4$, 1; D-glucose, 15; ascorbate, 2; (-)-bicuculline methochloride, 0.01, bubbled with O_2:CO_2: 95:5%. Visually-guided, whole-cell recordings were obtained at room temperature from the soma of CA1 neurons using patch electrodes that contained

(mM): $CsMeSO_4$, 130; HEPES, 10; NaCl, 8; EGTA, 0.5; Mg-ATP, 4; Na-GTP, 0.3; QX-314, 5. Schaffer collateral-commissural fibres were stimulated at a frequency of 0.1 Hz and excitatory postsynaptic current (EPSC) amplitude and access resistance recorded on-line at a holding potential of -70 mV. To induce NMDAR-dependent LTD, 300 pulses (at 0.66 Hz) at -40 mV, were delivered 20 to 40 min after formation of the whole-cell configuration. Under control conditions this usually induced a robust, homosynaptic NMDAR-LTD (Peineau et al., 2009; Peineau et al., 2007). Provided LTD was induced in the controls, experiments were interleaved in which various kinase inhibitors were included in the patch solution. Data were stored and analysed using the WinLTP Program (Anderson and Collingridge, 2007) and are presented as mean ± SEM. The magnitude of LTD was determined by comparing the average amplitude of responses over a 5 min period obtained immediately before and at least 20 min following the LTD induction protocol. To compare the magnitude of LTD in the different conditions, a non-parametric one-way ANOVA was performed. Significance was set at $P < 0.05$.

Whole-cell voltage-clamp recordings on organotypic slices were made from transfected CA1 pyramidal cells at 6-11 days *in vitro* and were performed blind with respect to the transfected plasmid. The extracellular solution contained (mM): NaCl, 124; KCl, 3; $NaHCO_3$, 26; NaH_2PO_4, 1.4; $CaCl_2$, 4; $MgSO_4$, 4; glucose, 10; picrotoxin, 50μM and 2-chloroadenosine, 1 μM, bubbled with O_2:CO_2: 95:5%. Two stimulating electrodes (test and control input) were placed in the Schaffer collateral-commissural pathway and stimulated at 0.05 Hz to record AMPAR EPSCs (V_h = -70 mV). To measure NMDAR EPSCs, neurons were held at +40 mV and the EPSC amplitude was measured 60 ms following the stimulus. NMDAR-LTD was induced using a pairing protocol (1 Hz for 6 min, V_h = -40 mV). Access resistance was monitored constantly and neurons were discarded if this varied by more than 20% during the recording period.

Biochemistry

Stimulation and western blot

For chemically-induced LTD, whole hippocampal slices (without CA3) were treated with either 20 μM NMDA for 3 min, 100 μM DHPG for 10 min or 50 μM carbachol for 10 min. For LFS-induced LTD, hippocampal slices were stimulated (900 stimulations at 1 Hz) with two electrodes placed in the Schaffer collateral-commissural fibres. The stratum radiatum (SR) surrounding the stimulating electrodes, enriched in CA1 dendrites, and the stratum pyramidale (SP), enriched in CA1 cell bodies, were then microdissected

within the next 10 min and washed in a cold buffer containing (mM): Tris, 20; NaCl, 150; EDTA, 5; EGTA, 1; NaF, 5 and Na_3VO_4, 1. The SR was lysed in the same buffer with 1% protease inhibitor (Roche Products, Welwyn Garden City, UK) and 1% Triton X-100 added. The SP was lysed in a sucrose buffer containing 11% sucrose, 10 mM HEPES, 5 mM NaF, 1% phosphatase inhibitor mixture 2 (Sigma-aldrich, St. Louis, MO) and 1% protease inhibitor. The lysates were homogenized with a pellet pestle and rotated for 30-40 min at 4°C. The SR was then centrifuged at 1,000 x g for 10 min and the supernatant was kept. The SP was centrifuged at 800 x g for 10 min and the pellet was washed once and passed through a 25G needle before a last centrifugation at 800 x g. Twenty µg of protein from these samples were denaturated at 95ºC for 5 min in a standard denaturating buffer, separated with SDS-PAGE and transferred onto a PVDF membrane (Bio-Rad Laboratories, Hercules, CA). Membranes were incubated for 1 h in TBS-Tween 1% (TBS-T) with 3% bovine serum albumin (BSA, Sigma-Aldrich). The antibodies used were: rabbit anti-phospho-JAK2 (Tyr 1007-1008, 09-275, Millipore, Billerica, MA, 1:500), rabbit anti-JAK2 (3230, Cell Signaling Technology, Danvers, MA, 1:500), mouse anti-phospho-STAT3 (Tyr 705, sc-8059, Santa Cruz Biotechnology, Santa Cruz, CA, 1:100), mouse anti-STAT3 (sc-8019, Santa Cruz Biotechnology, 1:100), mouse anti-β-actin (ab6276, abcam, Cambridge, MA, 1:10,000), rabbit anti-GAPDH (2118, Cell Signaling Technology, 1:10,000), rabbit anti-lamin (2032, Cell Signaling Technology, 1:200) and mouse or rabbit secondary antibodies (Millipore, 1:10,000 on TBS-T BSA 3%).

The intensity of the bands was quantified using WCIF ImageJ (NIH) software. For each experiment, the ratio of phosphorylated protein over the total amount of protein was calculated in each condition and compared relative to the ratio obtained in control condition. Results are presented as mean ± SEM. A paired Student's t-test was then performed. A value of $p < 0.05$ was considered significant.

Subcellular fractionation

Rat hippocampi were Dounce homogenized with 0.32 M sucrose solution containing both a protease inhibitor cocktail (Roche) and a phosphatase inhibitor cocktail I, II (Sigma). The homogenized suspension was centrifuged twice at 1,400 x g for 10 min at 4 °C to remove intact cells and large cellular organelles. Note that the pellet from the first spin was resuspended in the sucrose buffer and pelleted again. The obtained supernatants were combined and centrifuged at 10,000 x g for 20 min at 4 °C resulting in a supernatant and a second pellet (P2). The P2 pellet was resuspended in 0.32 M sucrose solution and layered over a sucrose step gradient (0.85/1.0/1.2M). The tube was

centrifuged at 82,500 x g for 2 h. The fraction between 1.0 M and the 1.2 M sucrose layers was collected and centrifuged at 17,000 x g to pellet the synaptosomal fraction. The pellet (LP1 fraction) was then further solubilized in a modified RIPA buffer (50 mM Tris-HCl, 150 mM NaCl, 2 % NP-40 alternative (Sigma), 0.5% sodium deoxycholate, 0.1% SDS, 1 mM EDTA with a protease inhibitor cocktail) for 30 min rotating at 4 °C and pelleted again to remove the remaining insoluble protein and lipid aggregates from the supernatant. Approximately 40 μg of each sample was resolved by SDS-PAGE and the membrane probed for PSD-95 (Affinity BioReagents; 1:1,000) and JAK2 (1:500) overnight at 4°C.

Hippocampal cell cultures

For STAT3 translocation to the nucleus, hippocampi were dissected from 18 day old embryo Sprague Dawley rat brains and dissociated in Hank's balanced salt solution (HBSS) with 0.25% trypsin and 0.1% DNAse I. Hippocampal neurons were plated on glass coverslips previously coated with gelatin and poly-L-lysine. Neurons were grown in Neurobasal Medium (Invitrogen, Carlsbad, CA) supplemented with B27 and glutamine (Invitrogen) and maintained in an incubator at 5% CO_2.

Pharmacological treatments: For time-course experiments, mature neurons (day *in vitro* 12) were treated with 20 μM D-serine (Ascent Scientific, Princeton, NJ) as control or 20 μM D-serine + 20 μM NMDA (Ascent Scientific) in Neurobasal medium for 10 min at 37°C. Neurons were then either fixed immediately (0 min) or fixed after rinsing and subsequent incubation in Neurobasal medium for 30, 60 or 120 min at 37°C.

In another sets of experiments, neurons were pretreated with 10 μM AG490 (Calbiochem) in Neurobasal medium for 30 min at 37°C, followed by incubation with either 20 μM D-serine alone or 20 μM D-serine + 20 μM NMDA in Neurobasal medium for 10 min at 37°C, and fixed immediately after.

To test the efficiency of the shRNAs and for the experiments with Stattic, hippocampi were dissected and dissociated from 2 day old Wistar rats.

Transfection of the cells with the shRNAs was performed at DIV 4-6 using lipofectamine 2000 according to the manufacturer’s protocol and the cells were fixed 2-3 days later. Pharmacological treatment with D-Serine and NMDA was performed as described above, 4-8 days after dissociation, on cells incubated with either control vehicle DMSO or Stattic (50 μM) for 20-30 min. Cells were then washed and lysed in a standard lysis buffer as described previously.

Immunocytochemistry

P-STAT3 and STAT3 immunoreactivity

Neurons were fixed with 4% paraformaldehyde (PFA) + 4% sucrose in 0.1 M phosphate buffer, pH 7.4 (PB) for 20 min at room temperature (RT). For P-STAT3 immunoreactivity, PFA fixed cells were rinsed in 0.01 M phosphate-buffered saline, pH 7.4 (PBS), and permeabilized with methanol at -20°C for 10 min. For both P-STAT3 and STAT3 detection, neurons were rinsed in PBS, and incubated 30 min with 5% normal donkey serum (NDS; Sigma-Aldrich) in PBS with 0.1% Triton X-100. Neurons were then incubated in either a rabbit polyclonal P-STAT3 (Tyr 705) antibody (9131; Cell Signaling Technology, Danvers, MA; 1:100) or with a mouse monoclonal STAT3 antibody (124H6; Cell Signaling Technology; 1:400) diluted in PBS with 1% NDS and 0.1% Triton X-100 overnight at 4°C. Neurons were rinsed 3 times in PBS, and incubated in Alexa Fluor 488-conjugated donkey anti-rabbit or donkey anti-mouse IgG, respectively (both 1:200; Molecular Probes, Eugene, OR) diluted in PBS with 1% NDS and 0.1% Triton X-100 for 60 min at RT. Finally, cells were rinsed in PBS, stained for a few seconds with DAPI (1:1,000), rinsed and mounted with Fluoromount (Southern Biotech, Birmingham, AL) for microscopic observations.

Neurons were imaged using a Zeiss Axio Observer inverted microscope (Carl Zeiss, Jena, Germany) equipped with an AxioCam MRm camera. Illumination and filter settings were maintained at the same level for image acquisition for all specimens. For each cell, signal intensity was measured over the nucleus and in the cytoplasm in uncompressed 8-bit gray scale tiff files using ImageJ 1.410 software (NIH), in an average of 10 cells/group from 3 independent experiments. Values were analysed by ANOVA followed by Dunnett's multiple comparison test for the time-course experiments and by Newman-Keuls multiple comparison test for the JAK2 inhibitor experiments. The value of $p < 0.05$ was considered statistically significant. Statistical analysis and building of graphs were performed using GraphPad Prism (version 5.00 for Windows; GraphPad Software, San Diego, CA).

JAK2 immunoreactivity

Mature neurons (day *in vitro* 12) were fixed with methanol at -20°C for 10 min. The cells were then washed in PBS and incubated for 30 min with 5% NDS in PBS with 0.1% Triton X-100. A rabbit polyclonal antibody was used to detect JAK2 (sc-278; Santa Cruz Biotechnology, Santa Cruz, CA; 1:50). Mouse monoclonal antibodies were used to

detect microtubule associated protein 2 (MAP2) (ab11268; Abcam Inc., Cambridge, MA; 1:1,000) and PSD-95 (05-494; Upstate Biotechnology, Billerica, MA; 1:200). Mixtures of primary antibodies were diluted in PBS with 1% NDS and 0.1% Triton X-100 and incubated overnight at 4°C. The following day, neurons were rinsed three times in PBS, followed by a 60 min incubation in a mixture of appropriate secondary antibodies diluted in PBS with 1% NDS and 0.1% Triton X-100. Secondary antibodies used were Alexa Fluor 488-conjugated donkey anti-rabbit (1: 200; Molecular Probes) and cyanine 3-conjugated donkey anti-mouse (1:300; Jackson ImmunoResearch, West Grove, PA). Finally, cells were washed in PBS and mounted with Fluoromount (Southern Biotech) for confocal microscopic analysis.

Neurons were analysed using a Zeiss Axio Observer inverted microscope equipped with a LSM 5 Exciter confocal scanning system (Carl Zeiss). To assess the extent of colocalisation between JAK2 and PSD-95 immunoreactivity, single optical sections were acquired in sequential mode and analysed using the LSM 5 Exciter software (Carl Zeiss). JAK2- and PSD-95-immunoreactive clusters with an area >0.2 μm^2 were identified and counted in the two channels separately, and those in the same position were considered as double-labeled. A total of 852 PSD-95- and 690 JAK2-immunoreactive clusters were analysed from 20 dendritic trees.

To build and label the composite illustrations, Adobe Photoshop (version 10.0.1) and Adobe Illustrator (version 13.0.0; Adobe Systems, San Jose, CA) were used.

JAK2 and STAT3 knockdown

Neurons were fixed with 4% PFA, washed with PBS and blocked with donkey serum as described previously. Neurons were then incubated with either STAT3 anbibody (as above) or JAK2 antibody (1:200, ab39636, abcam, Cambridge, MA) in 1% NDS and 0.1% triton. The secondary antibodies used were cyanine 3-conjugated donkey anti-rabbit (1:500; Jackson ImmunoResearch, West Grove, PA) or Alexa-594 donkey anti-mouse (1:1000, Invitrogen, Carlsbad, CA). Confocal images of transfected neurons were obtained with sequential acquisition on a Leica AOBS SP2 confocal imaging system attached to Leica DMIRE2 inverted microscope. Each image is a z-series of 10 images each averaged 4 times. The resulting z-stack was 'flattened' into a single image using maximum projection.

HEK cells and transfection

HEK293 cells were maintained in Dulbecco’s Modified Eagle’s medium supplemented

with 10% dialysed horse serum and 2 mM l-glutamine in a humidified incubator at 37 °C with 5% CO2. They were transfected with a pcDNA3-rJAK2(FL)-HA plasmid and the different shRNAs, using an Amaxa Nucleofector Kit V according to the manufacturer's instructions, after which 0.5 x 10^6 per well of transfected cells were plated on 6-well plates previously coated with poly-L-lysine and collagen. The cells were lysed in a standard lysis buffer, 72h after transfection, and the levels of JAK2 and GAPDH were analysed by western-blot.
